# Enhanced Functional Connectivity between Putamen and Supplementary Motor Area in Parkinson’s Disease Patients

**DOI:** 10.1371/journal.pone.0059717

**Published:** 2013-03-26

**Authors:** Rongjun Yu, Bo Liu, Lingling Wang, Jun Chen, Xian Liu

**Affiliations:** 1 School of Psychology and Center for Studies of Psychological Application, South China Normal University, Guangzhou, China; 2 Department of Image, Guangdong Provincial Hospital of Traditional Chinese Medicine, Guangzhou, China; Yale University School of Medicine, United States of America

## Abstract

Parkinson’s disease (PD) is a surprisingly heterogeneous disorder with symptoms including resting tremor, bradykinesia and rigidity. PD has been associated with abnormal task related brain activation in sensory and motor regions as well as reward related network. Although corticostriatal skeletomotor circuit dysfunction is implicated in the neurobiology of Parkinson’s disease, the functional connectivity within this circuit at the resting state is still unclear for PD. Here we utilized resting state functional magnetic resonance imaging to measure the functional connectivity of striatum and motor cortex in 19 patients with PD and 20 healthy controls. We found that the putamen, but not the caudate, exhibited enhanced connectivity with supplementary motor area (SMA), using either the putamen or the SMA as the “seed region”. Enhanced SMA-amygdala functional connectivity was also found in the PD group, compared with normal controls. Our findings highlight the key role of hyper-connected putamen-SMC circuit in the pathophysiology of PD.

## Introduction

Parkinson’s disease (PD) is a progressive neurological disorder characterized by tremor, rigidity, and slowness of movements, and is associated with progressive neuronal loss dopamine-generating cells in the substantia nigra and other brain regions. It has been shown that the dopamine uptake is reduced in the striatum, and the most severely affected region is the putamen [1]. Unilateral pallidotomy improves bilateral motor symptoms of Parkinson’s disease, especially for dyskinesias [2], [3]. The effective connectivity between the putamen and primary motor cortex (PMC), supplementary motor area (SMA), and cerebellum negatively correlated with the Unified Parkinson’s Disease Rating Scale (UPDRS) motor scores dysfunction of the putamen also correlates with clinical scores of akinesia [4]. These findings suggest that the putamen is primarily associated with motor dysfunction in Parkinson’s disease.

In healthy subjects, the putamen and the motor regions are functionally integrated during motor task preparation and execution. During the resting state, in order to be ready to perform a future motor task, the putamen-motor network must maintain in a dynamic equilibrium [5], [6]. A disrupted pattern of interactions of the motor network in the resting state is likely to cause observable motor deficits, e.g. akinesia, in PD. During execution of the motor task, the putamen exhibited stronger connectivity with controlling motor regions in both hemispheres [7]. The increased functional connectivity in this network may also reflect the primary pathophysiology of PD, e.g. tremor, due to an inability to inhibit contextually inappropriate circuits. Thus, normal daily-life movements may be difficult to achieve in Parkinson’s disease, because of the defective functional connectivity between the putamen and motor areas.

Using task-based functional magnetic resonance imaging (fMRI), previous studies have found that the supplementary motor area (SMA) and putamen are hypoactivated, whereas other cortical regions, like the cerebellum, premotor area (PMA), and parietal cortex are hyperactivated in patients with PD compared to normal subjects during performing motor tasks [4], [8], [9], [10]. However, the different activity observed between the patients with PD and normal subjects maybe due to changes in neural activity at the “task” state or at the resting state. Using positron emission tomography (PET), or single-photon emission computed tomography (SPECT), it has been revealed that regional metabolism in the resting state is abnormal in PD. The patients have hypermetabolism in some regions, like the globus pallidus, thalamus, and cerebellum, as well as hypometabolism in some other areas including prefrontal cortex, SMA, and parietal cortex [11], [12], [13]. Thus, it is likely that FC in the resting state is also different in PD and normal subjects, and these differences may contribute to the observed higher or lower activity in patients with PD detected by fMRI during task performance. Here, we used resting state functional magnetic resonance imaging (rs-fMRI) to investigate connectivity of the corticostriatal skeletomotor circuitry in Parkinson’s disease.

## Materials and Methods

### Participants

We studied 19 PD patients (11 men, aged 59.5±11.1 years; mean ± SD), and 20 age- and sex-matched healthy subjects (11 men, aged 59.2±8.7 years; mean±SD, also see Table 1). The distributions of age and gender were not significantly different between the two groups (P>0.37 and P>0.72, respectively). Patients were scanned after their medication had been withdrawn for at least 12 h. The subjects were all right-handed. PD was diagnosed based on medical history, neurological examinations, and MRI scans to exclude other central nerve system diseases. In the current study, all patients had an obvious at least a mild tremor. Patients were assessed with the UPDRS (Unified Parkinson’s Disease Rating Scale) while off their medications [14]. Exclusion criteria included the presence of DSM-IV Axis I diagnoses of other disorders such as bipolar disorder, history of any substance dependence or history of clinically significant head trauma,and claustrophobia. Exclusion criteria of head movement greater than 3 mm and rotation greater than 3 degrees during fMRI scanning resulted in the elimination of one subject in the Parkinson’s group. Participant motion parameters during fMRI scanning were shown in Table 1. There was no significant difference between two groups in all 6 motion parameters (p values >0.05). All of the controls are free of the DSM-IV diagnoses of schizophrenia and other DSM-IV Axis I diagnoses of other disorders. None of them has neurological diseases, history of any substance dependence, or history of clinically significant head trauma. The experiments were approved by the Institutional Review Board of the Guangdong Provincial Hospital of Traditional Chinese Medicine. All subjects gave their written informed consent for the study. All participants gave written, informed consent. They were informed of their right to discontinue participation at any time. All potential participants who declined to participate or otherwise did not participate were eligible for treatment (if applicable) and were not disadvantaged in any other way by not participating in the study.

**Table 1:** Sample Demographics.

Measure	PD (n = 19)		HC (n = 20)		statistics
	Mean	SD	Mean	SD	P
Age (year)	59.5	11	59.2	8.7	0.37
Gender (male)	11	n.a.	11	n.a.	0.72
Illness duration (year)	2.7	1.9	n.a.	n.a.	n.a.
UPDRS	26.8	13.4	n.a.	n.a.	n.a.
X (mm) Motionparameters	0.0258	0.149	−0.001	0.092	0.5
Y(mm)	0.039	0.120	0.007	0.137	0.4
Z(mm)	−0.099	0.190	0.029	0.213	0.07
Pitch (°)	−0.011	0.217	−0.041	0.220	0.68
Roll (°)	−0.068	0.261	−0.022	0.134	0.48
Yaw (°)	0.026	0.182	−0.002	0.075	0.56

Note Demographic information for the patient sample and control sample. Mean and standard deviation are provided for continuous variables (e.g., age, education, and UPDRS scales). PD = Parkinson’s disease. HC = healthy controls. UPDRS = unified Parkinson’s disease rating scale. Motion parameters - six motion parameters (translation: x, y and z in mm; rotation: pitch, roll and yaw in degrees) were obtained from head movement correction for each participant.

### MRI Data Acquisition

All subjects underwent structural and functional MRI scan in a single session using a 1.5T MR system (Magnetom Avanto Tim, Siemens, Germany). Sponges were used to fix subjects head within the coil to prevent motion artifact. All images were acquired parallel to anterior-commissure-posterior-commissure line with an auto-align technique. The resting state fMRI was performed with a gradient-echo echo planar sequence. Subjects were asked to relax and think of nothing in particular with eyes closed but were requested not to fall asleep. Wakefulness was assessed by directly asking them about their wakefulness after the resting state scanning via intercom link to the scanner chamber. The fMRI acquisition parameters were as follows: TR/TE = 2000 ms/24 ms, field of view (FOV) = 256 mm×256 mm, matrix = 64×64, slice thickness = 3 mm, interleaved scanning, and flip angle = 90°. For each participant, thirty-four trans-axial slices with no gap were acquired to encompass the whole brain volume. The scan time of the resting-state fMRI was approximately 6 minutes.

### Image Pre-processing

**Figure 1:**
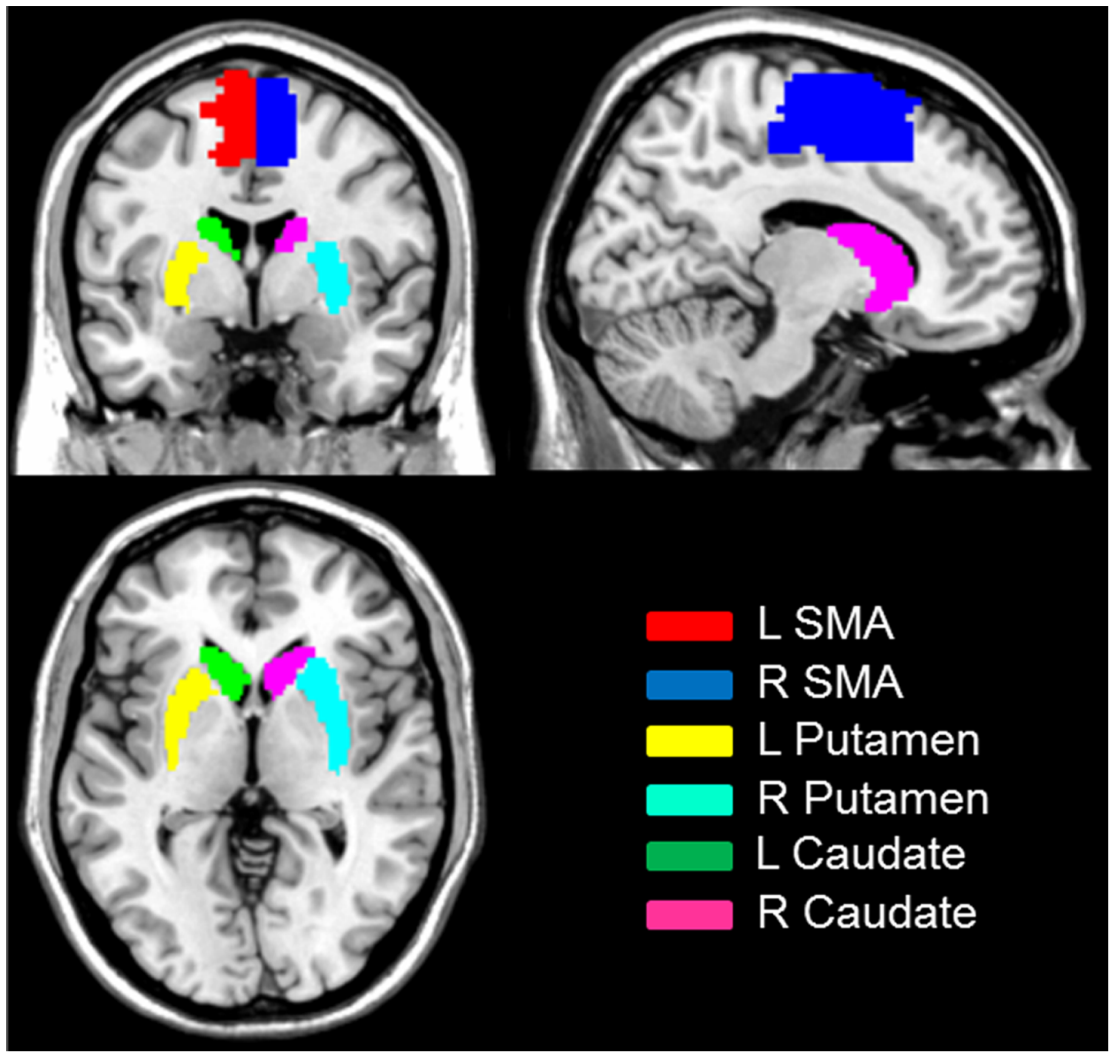
Seed regions for functional connectivity analysis.

The first 10 volumes of each functional time series were discarded because of instability of the initial MRI signal and adaptation of participants to the circumstance, leaving 170 volumes in total. The remaining fMRI images were slice acquisition corrected, head-motion corrected, normalized to the standard SPM5 Montreal Neurological Institute (MNI) template, and then re-sampled to 3-mm cubic voxels. After linear detrending, data was filtered using typical temporal bandpass (0.01–0.08 Hz) [15], [16]. Functional connectivity analysis was carried out by applying a seed-region approach using the left and right ROIs (putamen, caudate, and supplementary motor area, shown in **Figure 1**) as defined in the automated anatomical labeling atlas (AAL) [17]. For each ROI, individual participant time series statistical analyses were carried out using the General Linear Model (GLM) with the time series for the ROI, as well as for the nuisance covariates (whole brain signal intensity averaged across all brain voxels, white matter signal, cerebrospinal fluid, and six motion parameters) as predictors. These nuisance signals are typically adjusted for in resting-state functional connectivity studies because they reflect global signal fluctuations of nonneuronal origin (e.g., physiological artifacts associated with variables such as cardiac and respiratory cycles, CSF motion, and scanner drift) [18].

Contrast images were generated for each subject by estimating the regression coefficient (z value transformed from r value) between all brain voxels and each seed’s time series, respectively. One-sample t-test was used to identify positive and negative functional connectivity. For between group analysis, these images were then included in group (second-level) random effects analyses, adopting a 2×2 mixed design, factorial model (group [control, patient] by hemisphere [right seed, left seed]). To explore the correlations between FC and clinical scores, we used whole brain regression analysis with UPDRS and duration of illness as covariates of interest. A threshold of family-wise error (FWE) corrected threshold of p<0.05 using small volume correction (SVC) was set. The ROIs for svc include left putamen, right putamen, left caudate, right caudate, left SMA, right SMA, left amygdala, and right amygdala, defined in AAL [17]. These are key regions in the corticostriatal skeletomotor circuitry [7], [19]. Other activated brain areas were reported at a liberal threshold, P<0.001 uncorrected, 10 continuous voxels. All coordinates were reported in MNI coordinates, as used by SPM. To further control for the head motion effects, we also added six motion parameters as covariates into our regression models. Similar results were found after adding motor parameters in these models, suggesting that head motion cannot explain our functional connectivity findings.

## Results

### Putamen Seed

**Figure 2:**
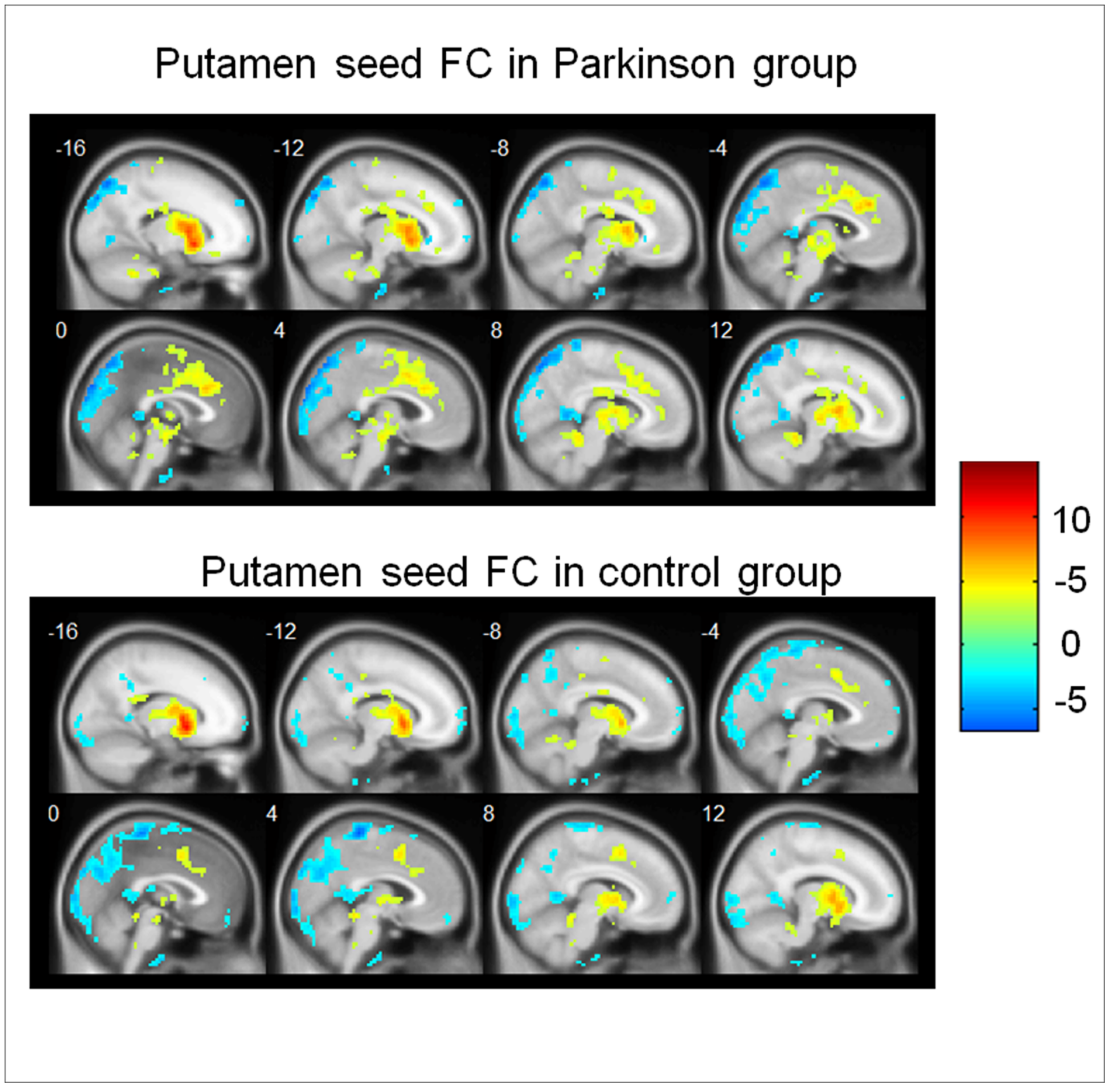
Functional connectivity with putamen in both groups. Regions that showed a significant functional connectivity (FC) with putamen in patients with Parkinson’s disease (upper panel) and in controls (lower panel). Hot color represents positive functional connectivity, whereas blue cold color represents negative functional connectivity. For display purposes only, all statistical maps (P<0.001, uncorrected) are overlayed on a T1-weighted MNI template.

Within-group functional connectivity patterns were revealed by one-sample t-tests in the PD group and control group, respectively (**Figure 2**). In the control group, some regions showed positive functional connectivity with left and right putamen-ROIs, including bilateral putamen, caudate, globus pallidus, midbrain, thalamus, insula, dorsal ACC and culmen. Negative functional connectivity with the putamen-ROIs in the controls was evident in some areas including bilateral postcentral Gyrus, precuneus, angular gyrus, superior parietal lobule, cuneus, middle temporal gyrus and declive. These findings are consistent with previous observations also using bilateral putamen as seeds [20]. The functional connectivity patterns of the PD group were roughly similar to that of the controls.

**Figure 3:**
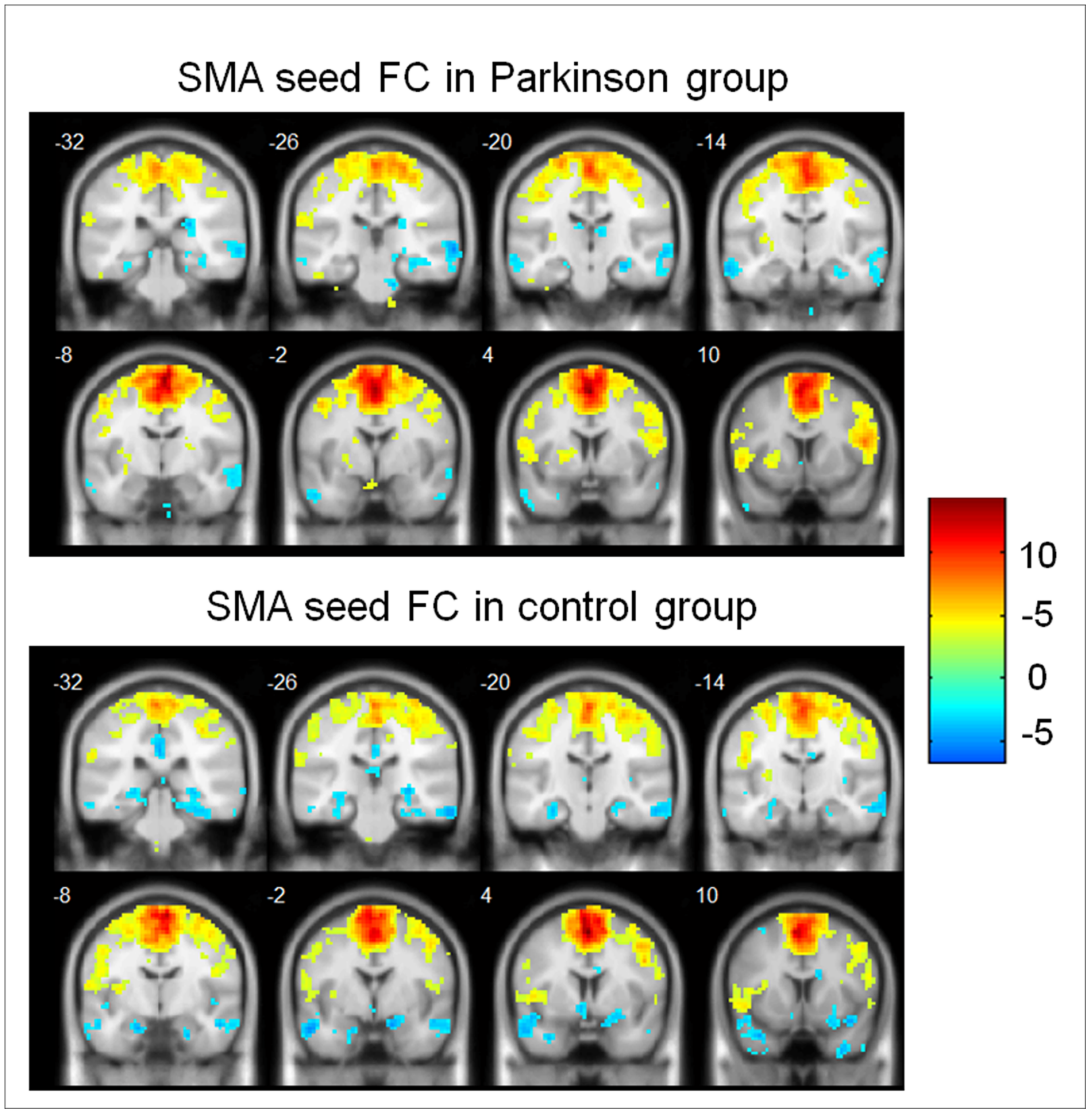
Functional connectivity with supplementary motor area in both groups. Regions that showed a significant functional connectivity (FC) with supplementary motor area (SMA) in patients with Parkinson’s disease (upper panel) and in controls (lower panel). Hot color represents positive functional connectivity, whereas blue cold color represents negative functional connectivity. For display purposes only, all statistical maps (P<0.001, uncorrected) are overlayed on a T1-weighted MNI template.

**Figure 4:**
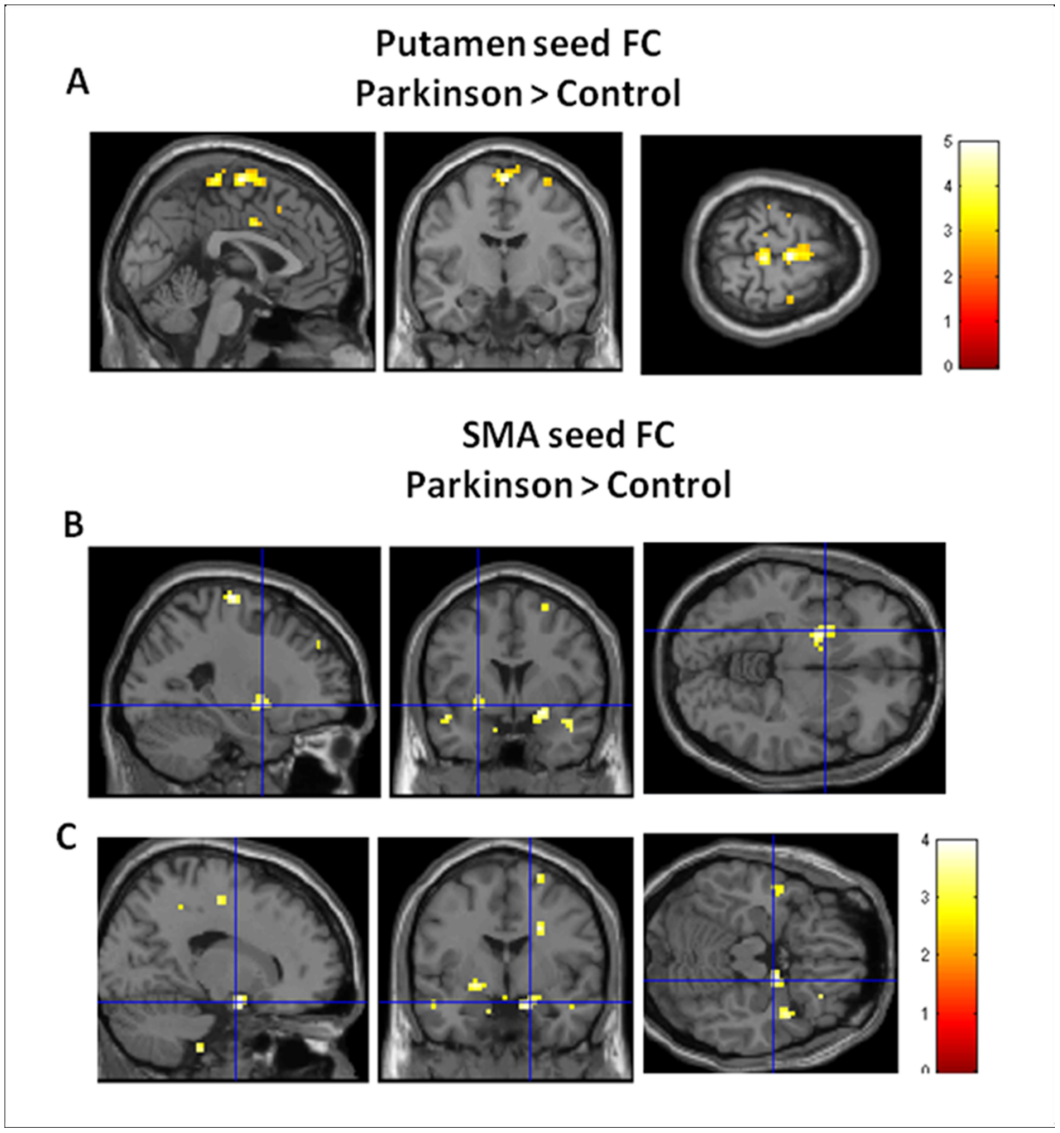
Group difference on functional connectivity. Regions that showed a significant stronger functional connectivity (FC) with putamen in supplementary motor area (SMA) (**A**) and stronger FC with SMA in left putamen (**B**) and right amydala (**C**) in patients with schizohprenia than controls. For display purposes only, all statistical maps (P<0.001, uncorrected) are overlayed on a T1-weighted MNI template.

**Figure 5:**
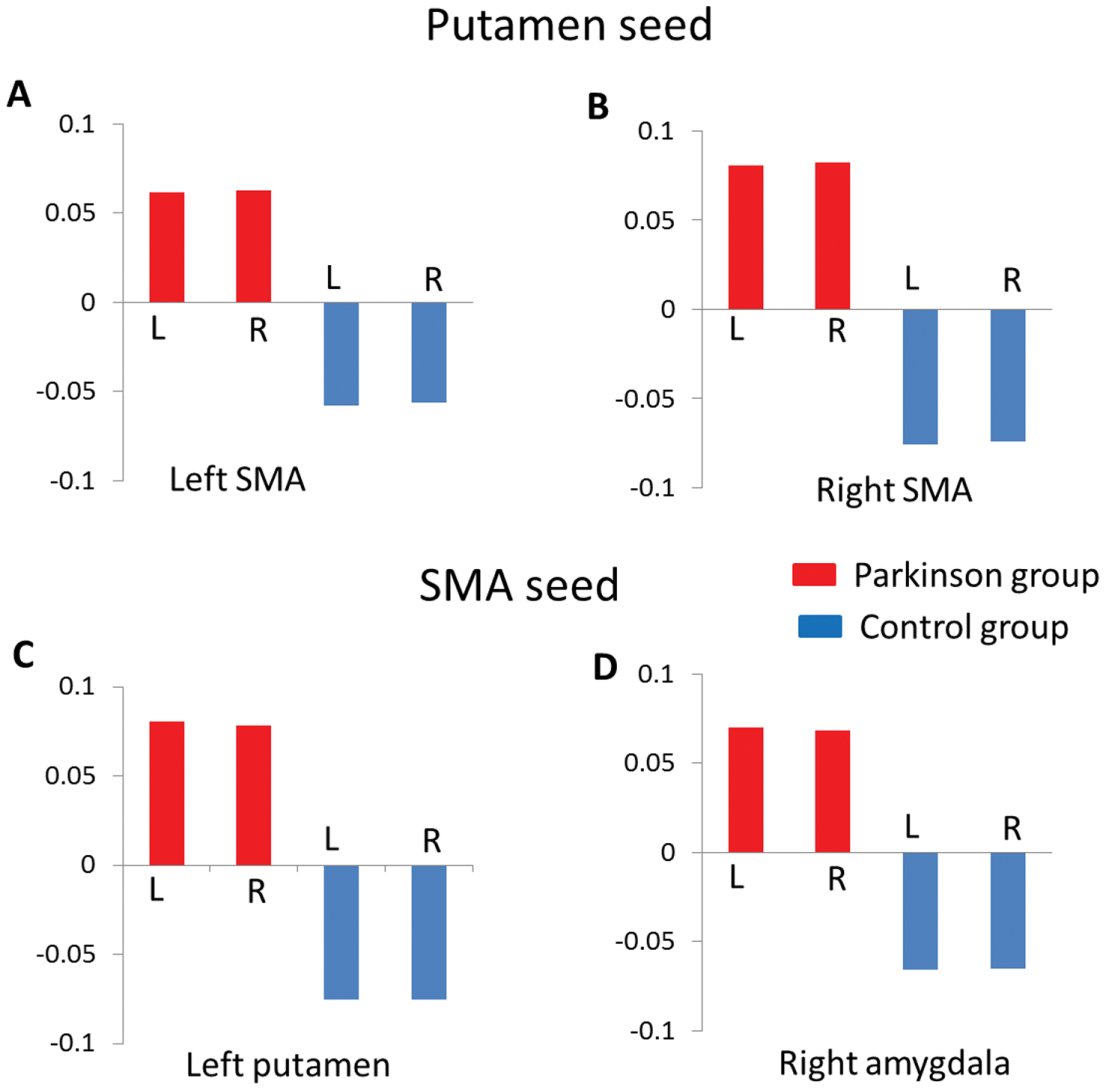
The beta values of functional connectivity in both groups. The patients group showed positive putamen-SMA functional connectivity, whereas the control group exhibited negative putamen-SMA functional connectivity (A&B). The patients group showed positive SMA-putamen (C) and SMA-amygdala (D) functional connectivity, whereas the control group exhibited negative functional connectivities. L: left seed; R: right seed.

The putamen seed based functional connectivity maps in both the PD group and the control group were shown in **Figure 3**. Compared to controls, patients exhibited enhanced FC with bilateral putamen in the supplementary motor area ([x = 0, y = −9, z = 69], peak Z = 4.34, voxel number = 44; [x = 3, y = −12, z = 69], peak Z = 4.60, voxel number = 27, svc P_FWE_<0.05, see **Figure 4A**). The putamen-SMA functional connectivity in Parkinson group was positive, whereas the putamen-SMA functional connectivity was negative in the control group (see **Figure 5A&B**). No significant BOLD signal increase was found for the opposite contrast (controls minus patients). No hemisphere by group interaction was found significant. No significant correlation with UPSRS was found.

### Caudate Seed

Compared to controls, patients exhibited reduced FC with bilateral caudate in the left OFC ([x = −21, y = 39, z = −6], peak Z = 3.66, voxel number = 40) only at a liberal threshold p<0.001 uncorrected. No significant activation was found for the opposite contrast (patients minus controls). No interaction was found significant. No significant correlation with UPSRS was found.

### Supplementary Motor Area Seed

The SMA seed based functional connectivity maps in both the PD group and the control group were shown in Figure 3. In the control group, some regions showed positive functional connectivity with left and right SMA-ROIs, including bilateral SMA, middle temporal gyrus precentral gyrus inferior frontal gyrus, superior frontal gyrus and insula whereas other regions like precuneus, cuneus, superior parietal lobe, posterior cingulate gyrus, parahippocampa gyrus, and ventromedial prefrontal cortex, exhibited negative functional connectivity with SMA [21]. Similar functional connectivity patterns were found in the PD group.

Compared to controls, patients exhibited enhanced FC with bilateral SMA in the left putamen ([x = −24, y = 3, z = −6], peak Z = 3.41, voxel number = 21, svc P_FWE_<0.05) and the right amygdala ([x = 18, y = −3, z = −18], peak Z = 3.37, voxel number = 7, svc P_FWE_<0.05), see Figure **4 B & C**. The SMA-putamen and SMA-amygdala functional connectivities in Parkinson group were positive, whereas both functional connectivities were negative in the control group (see **Figure 5 C&D**). No significant activation was found for the opposite contrast (controls minus patients). No interaction was found significant. No significant correlation with UPSRS was found.

## Discussion

In the present study, we examined changes in functional connectivity in the corticostriatal keletomotor circuit in PD. We found that patients with PD exhibited enhanced FC between SMA and bilateral putamen, but not between SMA and caudate. In addition, we also found exaggerated SMA-amygdala functional connectivity in PD. Our findings highlight the important role of putamen-SMA circuitry in the neuropathology of PD and suggest that the amygdala may also be associated with PD.

The putamen is the striatal nucleus primarily associated with motor performance, especially the “automatic” performance of previously learned movements. Studies in primate have found that putamen neurons were selective to the preprogrammed combination of movements and to the direction of the first movement [22], [23], [24]. Anatomically, the SMA is connected to the primary motor cortex, the premotor cortex, the primary somatosensory cortex, the striatum, and the thalamus [25].

The putamen-SMA connection is important for both self-initiated and externally triggered movements [26]. Some functional neuroimaging studies have investigated PD related functional connectivity changes in striatum and motor areas, but these observations were inconsistent as to whether there is stronger, or weaker, putamen-SMA functional connectivity in patients with PD [27], [28], [29]. For example, Wu et al. found reduced functional connectivity between SMA and the left putamen, right insula, right premotor cortex, and left inferior parietal lobule, in a group of PD with akinesia as the leading symptom. In their study, all patients had an obvious delay in movement initiation, but had at most a mild tremor. Kwak et al. only observed hyperconnectivity between putamen and cortical regions including ventromedial prefrontal cortex, anterior, and superior frontal gyrus [29]. No hypoconnectivity involving the putamen compared with controls was observed. The differences in sample characteristics, such as the stage of Parkinson’s disease, dominant symptoms, may contribute to the mixed findings. In our study, the enhanced connectivity with SMA is specific to the putamen but not in the caudate. This is consistent with previous resting state results showing that compared with the caudate, the putamen has stronger functional connectivity with the SMA [30].

Although it was not hypothesized, our data shows that the functional connectivity between SMA and the right amygdala is also enhanced in PD. Voon et al. (2010) described that patients with motor conversion disorder had greater functional connectivity between the right amygdala and the right supplementary motor area in response to emotional versus neutral stimuli [31]. Although there are no direct neuroanatomical projections between the amygdala and supplementary motor area, the amygdala projects to the striatum, which have projections via the pallidum and thalamus to the supplementary motor area [32]. The amygdala exhibits significant pathological changes in Parkinson’s disease, including atrophy and Lewy body formation and may contribute to some clinical features of PD [33]. It is worth mentioning that our study was not intended to specify the anatomic connections. The coactivation we report in the present study may be by way of either direct or indirect connections. Future studies may further investigate the role of amygdala in relation to motor deficits in the PD.

Some limitations in our study are worth mentioning. First, we did not examine the full spectrum of cognitive functions and social behaviours in our sample. Future investigation could focus on the relationships between FC alterations and function deficits. Second, the sample size in this study was moderate, which may limit the power to detect correlations between behavioural deficits and neural activity changes. Finally, the nature of the resting state functional connectivity and its relation to tasked based functional connectivity remains unclear. Future studies are necessary to investigate the biological and functional significance of FC at rest. Nevertheless, the consistency between regions found abnormal in PD using task-based fMRI and those identified in our R-fMRI suggest that R-fMRI can reveal meaningful and important neural network abnormalities. R-fMRI may have easier clinical applicability than standard fMRI, especially for patients like Parkinson’s disease who may not be able to perform certain tasks required in standard fMRI experiments due to their motor dysfunctions.

In conclusion, our study shows that putamen-SMA functional connectivity is enhanced in patients with PD. A more comprehensive understanding of the this specific network could lead to refinements of models of circuit pathology in movement disorders in Parkinson’s disease and help develop more effective treatments targeting this circutry.
