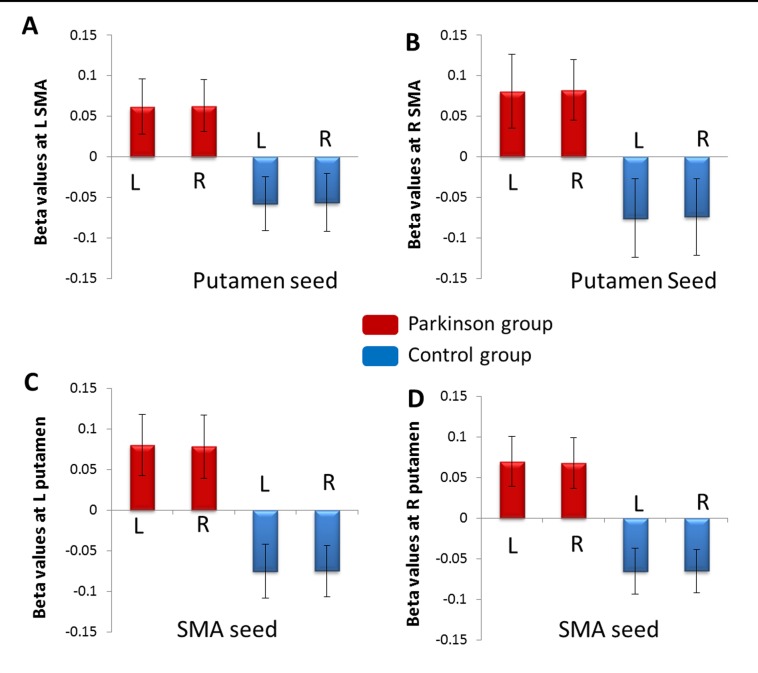# Correction: Enhanced Functional Connectivity between Putamen and Supplementary Motor Area in Parkinson’s Disease Patients

**DOI:** 10.1371/annotation/23b15701-3c35-49db-8dae-d4ef99e50d55

**Published:** 2013-10-23

**Authors:** Rongjun Yu, Bo Liu, Lingling Wang, Jun Chen, Xian Liu

Figure 5 is missing error bars. Please see the revised Figure 5 here: 

**Figure:**